# Are distinctive risk indicators associated with different stages of caries in children? A cross-sectional study

**DOI:** 10.1186/s12889-016-3865-4

**Published:** 2016-12-01

**Authors:** Maria Grazia Cagetti, Giovanna Congiu, Fabio Cocco, Gianfranco Meloni, Silvana Sale, Guglielmo Campus

**Affiliations:** 1 Department of Biomedical, Surgical and Dental Sciences, University of Milan, Milan, Italy; 2 WHO Collaboration Centre for Epidemiology and Community Dentistry, Milan, Italy; 3 Department of Surgery, Microsurgery and Medicine Sciences, School of Dentistry, University of Sassari, Viale San Pietro 43/C, I-07100, Sassari, Italy

**Keywords:** Dental caries, Children, Risk indicators, Caries staging, Feeding practice, Socio-economic status

## Abstract

**Background:**

Actual caries figures emphasize the need to identify the risk indicators involved in the disease’s development. The hypothesis that certain risk indicators might affect the dynamic evolution of the caries process was assessed; to clarify this premise, a cross-sectional survey was performed in school children.

**Methods:**

A total of 390 subjects aged 6–8 years old were randomly selected. Caries was assessed, and the subjects were stratified as follows: i) highest caries score; ii) most prevalent caries score; and iii) number of affected teeth. Parents/guardians completed a questionnaire regarding vital statistics, socio-economic indicators, dietary habits, oral hygiene habits and oral health behaviours.

**Results:**

Caries was detected in 42.31% of the subjects. Maternal nationality, parental education level, use of a sweetened pacifier at night, intake of lactose-free milk and toothbrushing frequency were statistically significant associated (*p* < 0.05) with subjects stratified according to the highest caries score. Parental educational level, maternal occupational status and use of a sweetened pacifier at night were associated (*p* < 0.05) with affected children stratified according to the most prevalent caries score. Maternal educational level and intake of lactose-free milk were associated with subjects with moderate caries stages compared to being caries-free (*p* = 0.01 and *p* = 0.02, respectively). Maternal nationality (*p* < 0.01) and toothbrushing frequency (*p* = 0.01) were associated with subjects affected by extensive lesions compared to caries-free children. In subjects affected by initial lesions as the most prevalent figure, gender (male) and paternal occupation status (unemployed) were statistically significant associated (*p* = 0.03 and *p* = 0.04, respectively) compared to those affected by highest prevalence of extensive caries lesions. In children with the highest prevalence of moderate caries lesions, maternal education level (*p* < 0.01), paternal occupational status (*p* = 0.03) and use of a sweetened pacifier at night (*p* < 0.01) were statistically significantly associated.

**Conclusions:**

Maternal nationality, maternal low level of education, intake of lactose-free milk and low toothbrushing frequency were involved in the change from caries-free status to different caries stages. Gender, paternal unemployment, maternal low educational level and use of a sweetened pacifier were correlated with caries progression, showing how distinctive risk indicators were associated with different caries stages.

**Electronic supplementary material:**

The online version of this article (doi:10.1186/s12889-016-3865-4) contains supplementary material, which is available to authorized users.

## Background

Although, in recent decades in Western countries, a decreasing caries trend has been observed, especially in childhood [1, 2], dental caries remains a major public health issue with impacts on the quality of life of children and adults [3]. Despite continuous improvements in dental health, children remain a primary disparity group [4]. Health risk evaluation is the comprehensive assessment of risk factors in the general population or specific groups, such as children, the elderly, subjects with immigrant backgrounds and so on, based on environmental, genetic, economic, social and behavioural health determinants [5]. The caries decrease has resulted in a polarization of the distribution of the disease, and today, high caries figures are observed only in a small proportion of the population [1, 4]. When the mean number of decayed, missing and filled teeth improves, the number of caries-free individuals increases, and polarization becomes more pronounced [6]. This skewed distribution emphasizes the need to identify caries risk indicators that are involved in the development of the disease to plan targeted preventive programmes that consider that health is linked to health promotion, particularly when care resources are limited [7].

Caries can be defined as a dietary-bacterial disease [8], with the interactions between the host and different risk factors derived from social inequalities, such as a low socio-economic level, recent immigrant status, a low educational degree and unemployment status [9, 10]. Considering the complex aetiology of the disease, a crucial issue is to identify its potential determinants and predictors to determine the appropriate public health measures to prevent it. Because caries lesions have a dynamic evolution requiring a long period of time, usually many months or years [11], it might be speculated that different risk indicators might be involved in several stages of the dynamic process, playing different roles at different times. This study began with the hypothesis that specific risk indicators might manifest their effects in specific caries stages, leading to distinct patterns that could be identified and studied. For example, subjects, especially children with initial lesions, should be considered at high risk for caries because these lesions are indicative of caries activity [12].

Another difficult issue is the complexity of the disease state because the different manifestations of caries cannot be quantified by a single metric. The progression of the lesion cannot be assessed with the most commonly used caries index, the DMFT/S index (the sum of decayed, missed, filled teeth/surfaces) because it does not distinguish among the different stages of caries lesions [13, 14]. The International Caries Detection Assessment System (ICDAS), which is based on the best available evidence for detecting the early and late stages of caries severity, should lead to the acquisition of better quality information to support decision-making at both the individual and community levels [14]. The ICDAS provides options for coding 6 different stages of caries lesions (0 sound, 1 first visual change in enamel, 2 distinct visual change in enamel, 3 localized enamel breakdown, 4 underlying dentin shadow, 5 distinct cavity with visible dentin, and 6 extensive cavity with visible dentin) [14].

As mentioned above, the premise of this study was to determine whether specific risk indicators might affect different stages of the caries disease process. To investigate this issue, a cross-sectional survey was designed and performed.

## Methods

### Sample selection and study design

**Fig. 1:**
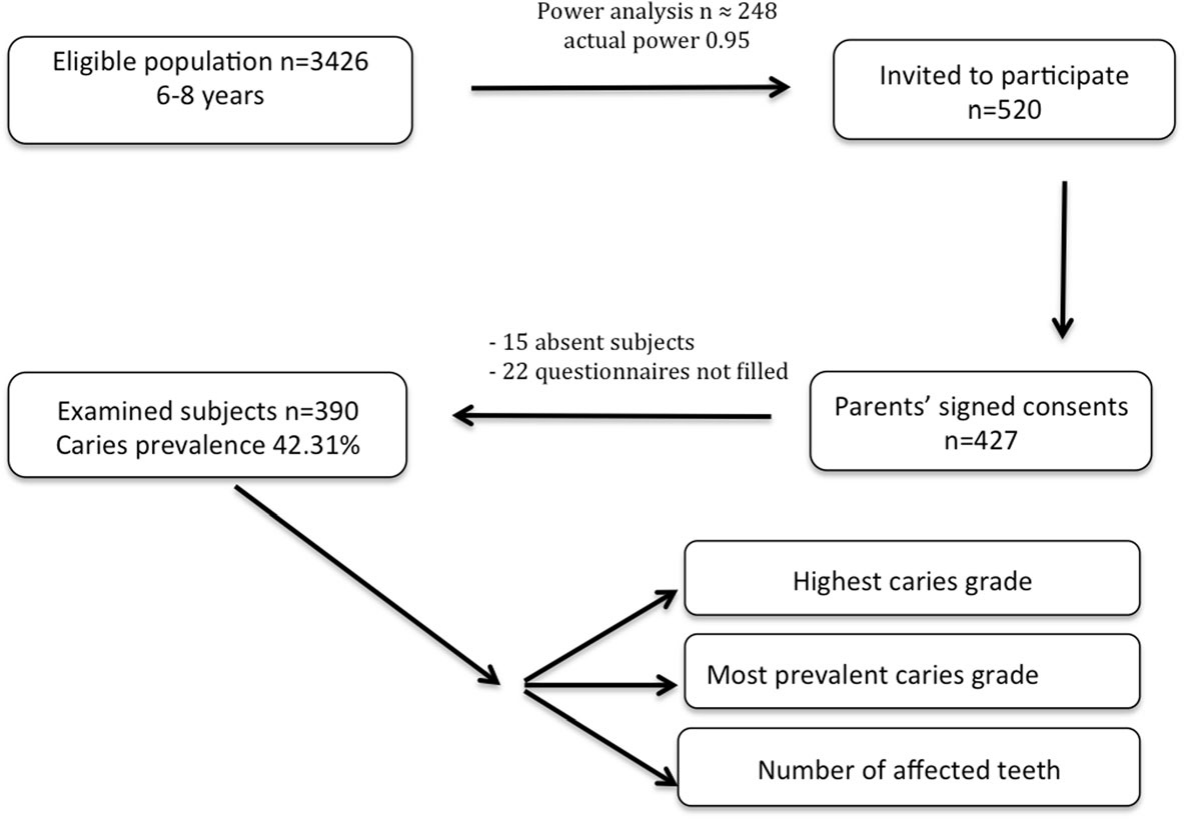
Flow diagram of the recruitment of children

The study protocol was approved by the Ethics Committee of the University of Sassari (registration no. SS0421/2012). The study was conducted in accordance with the tenets of the Declaration of Helsinki. According to data derived from the Italian Institute of Statistics [15] in 2013, the number of subjects aged 6–8 years old living in the Sassari area was 3095. The study used a cross-sectional design, and a formula for estimating population proportions with high precision was used to calculate the sample size. Data derived from the recent literature [16] described a prevalence of caries in the same age group of approximately 35%. A power analysis was performed with G*Power software, version 3.1.9.2 for Apple, using logistic regression, with the prevalence ratio added to 40% to safeguard against the risk of disease spread, an error probability of 0.05 and actual power of 0.95. The total sample size was set to 246. In February 2014, schoolchildren were recruited at their schools using systematic cluster sampling; every class was identified as a cluster, and the class list was compiled. The first cluster was randomly selected, while the others were systematically chosen at intervals of three classes. The number of subjects was approximately the same in each class (*n* = 20 subjects). Considering the possibility of a low response rate [17], 520 children were invited to participate through an information leaflet distributed to parents at the school, explaining the aim of the study and requesting their children’s participation. Only children with their parents’ signed consent were enrolled. Consent was obtained for 427 children. Parents were asked to complete a standardized questionnaire regarding different variables related to caries. Twenty-two subjects (5.15% dropouts) returned an incomplete questionnaire and were excluded from the survey; moreover, 15 (3.51%) children were absent from school on the day of the examination. The survey reported data from 390 children (Fig. 1).

### Data collection

The data collection methods consisted of a questionnaire and a clinical oral examination. A structured standardized questionnaire [18] was submitted to the parents/guardians before the examination. The questionnaire was based on the following domains: 1 - vital statistics: gender, parental nationality, and eruption timing of primary teeth; 2 - socio-economic indicators: parental educational level and parental occupational status; 3 - dietary habits: breast-feeding, bottle-feeding, sweet bottle-feeding at night, use of a sweetened pacifier at night, sweetened drinks before sleeping and use of lactose-free milk as the main diary product; 4 - oral hygiene habits: fluoride amount in toothpaste, frequency of toothbrushing, and fluoride supplement intake; and 5 - oral health behaviours: frequency of dental check-ups for the parents and children. As the clinical chart was printed in the other side of the sheet of questionnaire, the questionnaire form was collected directly by the examiner before the examination.

Children were examined at their schools using a calibrated examiner (GiC) from September to November of 2014. An author (GC) acted as a benchmark, training and calibrating the examiner (GiC). Fifty subjects, not included in the sample, were examined and re-examined after 72 h. Intra-examiner reproducibility was evaluated through percentage agreement and Cohen’s kappa statistics. The percentage agreement was high at both the subject and tooth surface levels (Cohen’s kappa = 0.86 and 0.77, respectively). Clinical examinations were performed under standardized conditions using a portable air-drier device, a plain mirror and a WHO Community Periodontal Index probe. Dental caries was assessed using the ICDAS II criteria and visual and tactile examinations (using only a ball-ended probe) [19, 20]; no radiographs were obtained [21].

### Data analysis

All of the data were input into a spreadsheet (Microsoft Excel® 2011 for Mac, version 14.4.3). Statistical analyses were performed using Stata/SE® software, version 13.1 for Mac (64-bit Intel®)(for raw data consultation please check Additional file 1).

Data from the clinical examinations were grouped as follows: No caries (ICDAS 0), Initial stage (ICDAS 1–2), Moderate stage (ICDAS 3–4) and Extensive stage (ICDAS 5–6). Regarding the number of affected teeth in each subject, the following categories were defined: 1–2 affected teeth, 3–5 affected teeth, and six or more affected teeth.

Subjects were stratified according to the following three different procedures: i) the maximum caries stage -- subjects were coded according to the highest caries score (ICDAS) recorded; ii) the most prevalent caries stage -- subjects were coded according to the most prevalent caries score (ICDAS) recorded; and iii) the number of affected teeth -- subjects were coded according to the number of affected teeth recorded. Responses to questionnaire items were treated as categorical or ordinal variables.

Associations between background variables and subjects, stratified as reported above, were assessed using the chi-squared test; for values less than five, Fisher’s exact test was performed.

Multinomial logit models were performed following the stratifying procedures to assess the associations with background variables. The multinomial logit model assumed that the data were case specific; each independent variable had a single value for each case. The model with the most severe caries grade as the dependent variable included the whole sample, using the caries-free subjects as the base outcome, while for the two other models, only caries-affected subjects were included, using the subjects with the most prevalent caries scores and the greatest number of affected teeth as the base outcome. An interaction model (likelihood statistics) tested the possible effects of modifiers of several background variables on the dependent variables.

Statistical significance was set as α < 0.05.

## Results

Three hundred ninety children were examined (51.45% male and 48.55% female). Caries lesions were detected in 42.31% of the subjects, while the caries experience (sum of decayed, filled and missing teeth due to caries) was 44.06%. According to the most severe caries lesion stage, the affected children were divided into 2.56% with initial stage, 13.33% with moderate stage and 26.42% with extensive stage. According to the most prevalent caries lesion stage, the affected children were divided into 4.10% with a prevalence of initial stage, 14.36% with a prevalence of moderate stage and the remaining 23.85% with a prevalence of extensive stage. According to the number of affected teeth, the children were divided into 18.21% with 1–2 affected teeth, 13.33% with 3–5 affected teeth and 10.77% with six or more affected teeth.

The sample distribution of the questionnaire items by gender is displayed in Table 1. Children with parents from countries of the European Union, a medium/low educational level and an occupational status as an employee accounted for most of the sample. More than 80% of the children appeared to have been breastfed, a few of them used fluoride supplements, and a non-negligible percentage (approximately 30%) does not use fluoride in their toothpaste either. Approximately one-third of the sample went to the dentist only in cases of pain, regarding both the children and parents.

**Table 1:** Distribution of the sample across questionnaire variables and gender

		Male *n (%)*	Female *n (%)*	Total *n (%)*
Parental nationality	European Union	177 (45.85)	175 (45.34)	352 (91.19)
	Not European Union	22 (5.70)	12 (2.11)	34 (8.81)
Maternal nationality	European Union	185 (47.56)	180 (46.27)	365 (92.90)
	Not European Union	15 (3.86)	9 (2.31)	24 (7.10)
Paternal nationality	European Union	189 (48.96)	181 (46.90)	370 (95.86)
	Not European Union	10 (2.59)	6 (1.55)	16 (4.14)
Eruption timing of primary teeth	<6 months	58 (15.22)	57 (14.96)	115 (30.18)
	6-9 months	124 (32.55)	117 (30.71)	241 (63.26)
	>9 months	16 (4.20)	9 (2.36)	25 (6.56)
Maternal educational level	Compulsory education	64 (16.45)	85 (21.85)	149 (28.30)
	Secondary school	104 (26.73)	82 (21.08)	186 (47.81)
	University	32 (8.23)	22 (5.66)	54 (13.89)
Paternal educational level	Compulsory education	99 (25.65)	110 (28.50)	209 (54.15)
	Secondary school	78 (20.20)	66 (17.10)	144 (37.30)
	University	22 (5.70)	11 (2.85)	33 (8.55)
Maternal occupational status	Housewife	69 (17.78)	78 (20.10)	147 (37.88)
	Unemployed	22 (5.67)	26 (6.70)	48 (12.37)
	Employed	89 (22.94)	59 (15.21)	148 (38.15)
	Self-employed	21 (5.41)	24 (6.19)	45 (11.60)
Paternal occupational status	Unemployed	29 (7.63)	25 (6.58)	54 (14.21)
	Employed	118 (31.05)	106 (27.90)	224 (58.95)
	Self-employed	52 (13.68)	50 (13.16)	102 (26.84)
Breast-feeding at least for 3 months	Yes	167 (42.93)	159 (40.87)	326 (83.80)
	No	34 (8.74)	29 (7.46)	63 (16.20)
Bottle-feeding at least for 3 months	Yes	164 (42.27)	152 (39.17)	316 (81.44)
	No	37 (9.54)	35 (9.02)	72 (18.56)
Sweet bottle-feeding at night	Yes	25 (6.63)	33 (8.75)	58 (15.38)
	No	167 (44.30)	152 (40.32)	319 (84.32)
Sweetened pacifier at night	Yes	57 (14.65)	73 (18.77)	130 (33.42)
	No	143 (36.76)	116 (29.82)	259 (66.58)
Sweetened drink before sleeping	No	70 (18.09)	67 (17.30)	137 (27.12)
	Yes	129 (33.33)	123 (31.26)	250 (64.60)
Lactose-free milk	Yes	23 (5.96)	16 (4.14)	39 (10.10)
	No	176 (45.60)	171 (44.30)	347 (89.90)
Fluoride content in toothpaste	Yes	142 (36.50)	125 (32.14)	267 (68.64)
	No	58 (14.91)	64 (16.45)	122 (31.36)
Frequency of toothbrushing	1/day	41 (10.54)	31 (7.97)	72 (18.51)
	2/day	110 (28.28)	116 (29.82)	226 (58.10)
	>2/day	50 (12.85)	41 (10.54)	91 (23.39)
Fluoride supplement	Yes	11 (2.87)	17 (4.44)	28 (7.31)
	No	185 (48.30)	170 (44.39)	355 (92.69)
Frequency of dental check-ups, parents	6 months	31 (8.20)	20 (5.29)	51 (13.49)
	Once per year	63 (16.67)	67 (17.72)	130 (34.39)
	Once every 2 years	33 (8.73)	29 (7.67)	62 (16.40)
	When in pain	68 (17.99)	67 (17.73)	135 (35.72)
Frequency of dental check-ups, children	6 months	34 (12.27)	23 (8.30)	57 (20.57)
	Once per year	51 (18.41)	49 (17.69)	100 (36.10)
	Once every 2 years	8 (2.89)	14 (5.06)	22 (7.95)
	When in pain	51 (18.41)	47 (16.97)	98 (35.38)

The associations among background variables derived from the questionnaires and the different stratifying procedures are reported in Tables 2 and 3. The most severe caries stage was statistically associated with a non-European Union maternal birthplace (*p* = 0.02), with the educational levels of both parents (*p* < 0.01) and with some behavioural habits, such as the use of a sweetened pacifier at night (*p* = 0.03), the use of lactose-free milk (*p* = 0.046) and the frequency of toothbrushing (*p* = 0.046). The distribution of affected children, according to the most prevalent caries lesion stages, showed statistically significant associations with parental educational level (*p* = 0.02 mother and *p* < 0.01 father), with the occupational status of the mother (*p* = 0.03) and with the use of a sweetened pacifier at night (*p* = 0.02). No statistically significant associations between the stratification number of affected teeth and background variables were found (*data not in table*).

**Table 2:** The distribution of the population across background variables according to the most severe caries stages

Questionnaire Items	Highest caries score (ICDAS)				
	Healthy n (%)	Initial n (%)	Moderate n (%)	Extensive n (%)	*p*-value
Maternal nationality					
European Union	217 (59.45)	9 (2.46)	47 (12.88)	92 (25.21)	
Not European Union	7 (29.17)	1 (4.17)	5 (20.83)	11 (45.83)	0.02
Parental educational level					
Mother					
Compulsory education	86 (57.72)	2 (1.34)	12 (8.05)	49 (32.89)	
Secondary school	107 (57.53)	5 (2.69)	30 (16.13)	44 (23.65)	
University	31 (57.41)	3 (5.55)	10 (18.52)	10 (18.52)	<0.01
Father					
Compulsory education	114 (54.55)	3 (1.44)	27 (12.92)	65 (31.09)	
Secondary school	89 (61.81)	3 (2.08)	22 (15.28)	30 (20.83)	
University	19 (57.58)	4 (12.12)	3 (9.09)	7 (21.21)	<0.01
Maternal occupational status					
Housewife	89 (60.54)	1 (0.69)	15 (10.20)	42 (28.57)	
Unemployed	24 (50.00)	2 (4.17)	6 (12.50)	16 (33.33)	
Employed	88 (59.46)	4 (2.70)	27 (18.24)	29 (19.60)	
Self-employed	24 (53.33)	3 (6.67)	3 (6.67)	15 (33.33)	0.06
Sweetened pacifier at night					
Yes	71 (54.61)	3 (2.31)	11 (8.46)	45 (34.62)	
No	153 (59.08)	7 (2.70)	41 (15.83)	58 (22.39)	0.03
Lactose-free milk					
Yes	16 (41.03)	1 (2.56)	10 (25.64)	12 (30.77)	
No	207 (59.65)	9 (2.59)	41 (11.82)	90 (25.94)	0.046
Frequency of toothbrushing					
1/day	37 (51.39)	3 (4.17)	7 (9.72)	25 (34.72)	
2/day	122 (53.98)	6 (2.65)	35 (15.49)	63 (27.88)	
> 2/day	65 (71.43)	1 (1.10)	10 (10.99)	15 (16.48)	0.046

The chi-square test was performed, and when a cell had a value less than five, Fisher’s exact test was performed

**Table 3:** The distribution of affected subjects, according to the most prevalent caries stages (initial/moderate/extensive) and background variables

Questionnaire items	Most prevalent caries stage			
	Initial *n (%)*	Moderate *n (%)*	Extensive *n (%)*	*p*-value
Maternal educational level				
Compulsory education	2 (3.17)	12 (19.05)	49 (77.78)	0.02
Secondary school	5 (6.33)	30 (37.97)	44 (55.70)	
University	3 (13.04)	10 (43.48)	10 (43.48)	
Paternal educational level				
Compulsory education	3 (3.16)	27 (28.42)	65 (68.42)	<0.01
Secondary school	3 (5.46)	22 (40.00)	30 (54.54)	
University	4 (28.57)	3 (21.43)	7 (50.00)	
Maternal occupational status				
Housewife	1 (1.73)	15 (25.86)	42 (72.41)	0.03
Unemployed	2 (8.33)	6 (25.00)	16 (66.67)	
Employed	4 (6.67)	27 (45.00)	29 (48.33)	
Self-employed	3 (14.29)	3 (14.29)	15 (71.43)	
Sweetened pacifier at night				
Yes	3 (5.08)	11 (18.64)	45 (76.28)	0.02
No	7 (6.60)	41 (38.68)	58 (54.72)	

The chi-squared test was performed, and when a cell had a value less than five, Fisher’s exact test was performed

The multinomial analysis is presented in Tables 4 and 5. The process of assessing the model showed no statistically significant effect modifiers on the dependent variables of the interaction between background covariates. The use of the most severe caries stage as a dependent variable showed that, in children with initial caries stages compared to those who were caries-free (considered as the base outcome), a frequency of toothbrushing of more than two times per day was near the significance level.

**Table 4:** Multinomial logistic regression

Number of observations = 390		log likelihood = −363.04		*p* < 0.01
		Coef (SE)	*p* > |z|	95% Conf. interval
Caries-free *(ICDAS = 0)*	Base outcome			
Initial lesions *(ICDS =1/2)*	Maternal nationality *(Not European Union)*	1.05 (1.15)	0.36	−1.19/3.29
	Maternal educational level *(Compulsory)*	0.59 (0.36)	0.11	−1.14/1.26
	Paternal occupational status *(Unemployed)*	0.66 (0.56)	0.24	−0.45/1.77
	Toothbrushing frequency *(1/day)*	−1.12 (0.56)	0.05	−2.22/−0.01
	Lactose-free milk *(Yes)*	0.27 (1.13)	0.81	−1.95/2.49
	Sweetened pacifier at night *(Yes)*	0.16 (0.70)	0.83	−1.31/1.63
Moderate lesions *(ICDS = 3/4)*	Maternal nationality *(Not European Union)*	0.93 (0.63)	0.14	−0.30/2.15
	Maternal educational level *(Compulsory)*	0.40 (016)	0.01	0.08/0.72
	Paternal occupational status *(Unemployed)*	−0.55 (0.28)	0.05	−1.08/−0.01
	Toothbrushing frequency *(1/day)*	−0.27 (0.25)	0.28	−0.77/0.22
	Lactose-free milk *(Yes)*	1.09 (0.46)	0.02	0.19/2.00
	Sweetened pacifier at night *(Yes)*	0.63 (0.39)	0.10	−1.40/0.13
Extensive lesions *(ICDS = 5/6)*	Maternal nationality *(Not European Union)*	1.39 (0.51)	<0.01	0.39/2.41
	Maternal educational level *(Compulsory)*	−0.10 (0.12)	0.39	−0.33/0.13
	Paternal occupational status *(Unemployed)*	0.03 (0.20)	0.86	−0.36/0.43
	Toothbrushing frequency *(1/day)*	−0.52 (0.20)	0.01	−0.91/−0.13
	Lactose-free milk *(Yes)*	0.49 (0.42)	0.24	−0.33/1.31
	Sweetened pacifier at night *(Yes)*	0.40 (0.26)	0.13	−0.11/0.91

The most severe caries stage (caries-free, initial, moderate, extensive) was used as the dependent variable

**Table 5:** Multinomial logistic regression in affected subjects

Number of observations = 165		log likelihood = −117.77		*p* < 0.01
		Coef (SE)	*p* > |z|	95% Conf. Interval
Initial lesions *(ICDS =1/2)*	Maternal educational level *(Compulsory)*	0.16 (0.41)	0.70	−0.64/0.95
	Paternal educational level *(Compulsory)*	0.77 (0.62)	0.22	−0.45/1.98
	Gender *(Male)*	1.77 (0.82)	0.03	0.16/3.39
	Paternal occupational status *(Unemployed)*	0.89 (0.45)	0.04	0.03/1.77
	Sweetened pacifier at night *(Yes)*	0.12 (0.84)	0.88	−1.52/1.76
Moderate lesions *(ICDS = 3/4)*	Maternal educational level *(Compulsory)*	0.60 (0.21)	<0.01	0.19/1.02
	Paternal educational level *(Compulsory)*	−0.19 (0.19)	0.34	−0.58/0.20
	Gender *(Male)*	−0.15 (0.38)	0.69	−0.89/0.59
	Paternal occupational status *(Unemployed)*	−0.71 (0.32)	0.03	−1.35/−0.08
	Sweetened pacifier at night *(Yes)*	−1.16 (0.43)	<0.01	−2.01/−0.31
Extensive lesions *(ICDS = 5/6)*	Base outcome			

The most prevalent caries stage (initial, moderate, extensive) was used as the dependent variable

In subjects with moderate caries stages, maternal educational level (compulsory level) and the use of lactose-free milk as the principal dairy drink were associated with caries-free subjects (coef = 0.40 and *p* = 0.01 and coef = 1.09, *p* = 0.02, respectively). Maternal nationality from outside the European Union (coef = 1.39, *p* < 0.01) and toothbrushing frequency (1 time/day) (coef = −0.52, *p* = 0.01) were associated in subjects with extensive lesions with regard to the base outcome (Table 4). In affected subjects (42.31% of the total sample examined), the most prevalent caries stage was used as the dependent variable. In this model, the subjects with prevalence of extensive caries stages were used as the base outcome (Table 5). In subjects with prevalence of initial lesions, gender (male) and paternal occupational status (unemployed) were statistically significant associated (coef = 1.77, *p* = 0.03 and coef = 0.89, *p* = 0.04, respectively) among the subjects with the prevalence of extensive caries stages. In the comparison between subjects with prevalence of moderate caries lesions and the base outcome (subjects with extensive caries stages), maternal education level (coef = 0.60, *p* < 0.01), paternal occupational status (coef = −0.71, *p* = 0.03) and the use of a sweetened pacifier at night (coef = −1.16, *p* < 0.01) were statistically significantly associated. Because no statistical associations were observed between the stratification number of affected teeth and the background variables, it was decided not to run a multinomial model.

## Discussion

The purpose of this study was to determine whether distinct caries risk indicators were associated with different stages of the caries disease process in a sample of schoolchildren in a cross-sectional survey. The results showed that several risk indicators interacted with disease evolution, mainly parental socio-economic and child behavioural indicators.

In this survey, caries distribution indicated a highly skewed figure with a large proportion of caries-free subjects, a small proportion of subjects with caries at the initial or moderate stage and a very large proportion of children with caries at extensive stages. A skewed distribution of the disease in young populations from Western countries indicates the actual caries figures [1, 2], and in Sardinia too [16], the prevalence of the disease was reported as quite low with a high percentage of caries-free children (64.5%).

Several questionnaire items were associated with the stratifications of children with regard to the dynamic evolution of the caries process. Considering the entire sample and according to the highest caries score, parental educational level was shown to be highly significantly associated. The socio-economic level of the family influences the health of all family members [22]. The knowledge and personal and social skills provided through education can better endow individuals to access, maintain and improve their own and their families’ health [23]. Higher levels of mothers’ education seemed to have a positive impact on children’s health [24] and on future health in adulthood [25]. Educational level remains quite stable after the school years and during adulthood, so it has a stable impact on health [25]. Considering the other socio-economic indicators, parental nationality and occupational status, only those factors related to the mother showed significant associations with the different caries stages. The mother’s educational level and occupational status were associated with both the highest caries score and the most prevalent score. As reported above, the educational level indicator has great stability and a large influence on behaviours and habits related to health. The father’s educational level was also demonstrated to be associated because both maternal knowledge and paternal knowledge affect children’s oral health. Among dietary habits and hygiene behaviours, only the use of a sweetened pacifier at night, the regular intake of lactose-free milk and the frequency of toothbrushing were significantly associated. Although the relationship between sugar and dental caries has become weaker in fluoridated societies, fermentable carbohydrates remain a primary factor in the development of caries, especially in children [26]. Sugar consumption among children has been associated with the mother’s level of education and household income [27].

A multinomial model in which caries-free subjects were used as the base outcome was used to evaluate the roles of diverse risk indicators able to change the status from caries-free to different caries stages. In subjects affected by initial caries, no variables played roles in the change of status, while in those with moderate caries, a low level of education of the mother and the intake of lactose-free milk as the primary dairy product were the covariates involved in the process. In children with the highest caries stage, maternal nationality outside the European Union and a low frequency of toothbrushing habits were involved. All of these findings emphasize the links between causal risk factors (intake of sugars and plaque) and socio-economic factors, mainly related to the mother (level of education and nationality).

Considering the caries-affected children stratified according to the most prevalent caries lesions stage, parental educational level, maternal occupational status and use of a sweetened pacifier at night were associated with this stratification. In the multinomial model, the base outcome was the subjects with prevalence of the highest caries stage; in children with a majority of initial lesions, gender (male) and the unemployment status of the father were embodied in this stage. In subjects with prevalence of moderate lesions, a low educational level of the mother, paternal unemployment status and use of a sweetened pacifier at night were the distinguishing factors. Socio-economic indicators and casual factors acted in synergy also in affected subjects, and as the lesions progressed, diet became increasingly important in the process.

No statistically significant associations of the categorization of the number of affected teeth and background variables were found, probably owing to the highly skewed caries distribution.

The results of this study must consider its weaknesses and strengths. Firstly, the cross-sectional nature of the data used in this analysis did not allow for the investigation of the directionality of the associations or the clarification of the time frames of the exposures. Nevertheless because some unalterable risk factors are involved in caries development, such as educational level in adulthood, as well as factors that are immutable over short periods of time, such as occupational status [10], these risk factors or “indicators” might also be investigated in cross-sectional studies. Secondly, caries is a continuous disease process, and the timing or the exposure time was not considered in the analysis. Otherwise, socioeconomic risk indicators act beginning at birth and tooth eruption, while causal factors might act at every moment of the subject’s life. Thirdly, the study considered only the associations between caries indicators and the actual disease (caries lesions) without considering caries consequences, such as filled and missing teeth due to caries. This limitation might have affected the associations with risk indicators; otherwise, the weight of the filled and missing component of caries experience in these age groups was quite low, as reported in the Results section. Regarding strengths, this survey started from the premise of evaluating risk indicator from a different point of view; usually, their roles are considered as a whole and not with consideration of the disease as a continuous process. In the literature, only one paper applied a similar approach, investigating the association between cavitated or non-cavitated caries lesions and background factors [28]. The sample examined was quite large and representative of the study population with the same age range, although the generalization of study results is limited to similar populations living in areas of Western countries with a medium/low *per capita* income, such as Sardinia.

## Conclusions

The results of the present paper, within the limitations described above, provided information about how distinctive risk indicators were associated with different caries stages. Maternal socio-economic indicators and children’s behaviours were involved in the changes from caries-free status to different caries stages. In the differentiation of initial from moderate and to severe caries stages, parental socio-economic indicators and oral health behaviours were embodied in caries progression. Socio-economic indicators and casual factors acted in synergy, and as the lesion progressed, diet became increasingly important in the process.

## Additional file

Additional file 1: Data set raw data. (XLSX 82 kb)

## Abbreviations

Coef: Coefficient
GC: Guglielmo Campus, the author who acted as benchmark for the calibration of the examiner
GiC: Giovanna Congiu, the author who was involved in the clinical examination
ICDAS: International Caries Detection Assessment System
WHO: World Health Organization
